# Exercise-induced circulating microRNA changes in athletes in various training scenarios

**DOI:** 10.1371/journal.pone.0191060

**Published:** 2018-01-16

**Authors:** Martin Horak, Filip Zlamal, Robert Iliev, Jan Kucera, Jan Cacek, Lenka Svobodova, Zuzana Hlavonova, Tomas Kalina, Ondrej Slaby, Julie Bienertova-Vasku

**Affiliations:** 1 Department of Pathological Physiology, Faculty of Medicine, Masaryk University, Kamenice 5, Brno, Czech Republic; 2 Research Centre for Toxic Compounds in the Environment, Masaryk University, Kamenice 5, Brno, Czech Republic; 3 Department of Athletics, Swimming and Outdoor Sports, Faculty of Sports Studies, Masaryk University, Kamenice 5, Brno, Czech Republic; 4 Central European Institute of Technology, Masaryk University, Kamenice 5, Brno, Czech Republic; University of Texas MD Anderson Cancer Center, UNITED STATES

## Abstract

**Background:**

The aim of the study was to compare selected extracellular miRNA levels (miR-16, miR-21, miR-93 and miR-222 with the response to 8-week-long explosive strength training (EXPL), hypertrophic strength training (HYP) and high-intensity interval training (HIIT).

**Methods:**

30 young male athletes of white European origin (mean age: 22.5 ± 4.06 years) recruited at the Faculty of Sports Studies of Masaryk University were enrolled in this study. The study participants were randomly assigned to three possible training scenarios: EXPL, HYP or HITT and participated in 8-week-long program in given arm. Blood plasma samples were collected at the baseline and at week 5 and 8 and anthropometric and physical activity parameters were measured. Pre- and post-intervention characteristics were compared and participants were further evaluated as responders (RES) or non-responders (NRES). RES/NRES status was established for the following characteristics: 300°/s right leg extension (t300), 60°/s right leg extension (t60), isometric extension (IE), vertical jump, isometric extension of the right leg and body fat percentage (BFP).

**Results:**

No differences in miRNA levels were apparent between the intervention groups at baseline. No statistically significant prediction role was observed using crude univariate stepwise regression model analysis where RES/NRES status for t300, t60, IE, vertical jump and pFM was used as a dependent variable and miR-21, miR-222, miR-16 and miR-93 levels at baseline were used as independent variables. The baseline levels of miR-93 expressed an independent prediction role for responder status based on isometric extension of the right leg (beta estimate 0.76, 95% CI: -0.01; 1.53, p = 0.052).

**Discussion:**

The results of the study indicate that 8-week-long explosive strength training, hypertrophic strength training and high-intensity interval training regimens are associated with significant changes in miR-16, mir-21, miR-222 and miR-93 levels compared to a baseline in athletic young men.

## Introduction

Regular physical activity has been known to influence health and the general quality of life in a beneficial manner, e.g. by preventing and reducing the risk of diseases including metabolic and age-related diseases and even cancer and by favorably affecting the mitochondrial metabolism as well as cognitive, cardiovascular and immune functions [1,2]. Furthermore, the specific training protocols have been known to affect distinct signaling pathways and thus influence different exercise-associated traits including angiogenesis, inflammation, muscle recovery, mitochondrial biogenesis, metabolic adaptations and many others. However, the molecular mechanisms of these physiological changes are not yet well defined and, to add to the complexity, are also prone to significant inter-individual variability. Nevertheless, variability in physical activity-induced adaptations may be partially explained by epigenetic factors such as non-coding RNAs.

MicroRNAs (miRNAs) are a group of endogenous short non-coding RNA molecules (approximately 18–23 nucleotides long) negatively regulating gene expression predominantly at the post-transcription level [3]. The expression of miRNAs frequently changes in a specific manner in response to various physiological or pathological conditions such as inflammation, cancer, cardiovascular disease, muscle hypertrophy and remodeling or exercise [4–8]. Moreover, miRNAs are released into circulation and other body fluids in a highly stable cell-free form which makes them excellent potential diagnostic or predictive biomarkers associated with the rate of change of specific conditions [9,10]. Researchers have therefore attempted to characterize the expression patterns of candidate miRNAs reflecting physical exercise adaptations on a molecular level in order to predict physical performance capacity, prevent muscle injury and monitor recovery. While many recent studies–listed and compared in several exercise-oriented reviews–have analyzed circulating miRNAs in response to acute or long-term physical training, their results remain inconclusive and generally unclear [6,11,12].

Currently, no reliable non-invasive physical fitness biomarkers assessing molecular and cellular events are available, which restricts any assessment of bodily response to a physical fitness evaluation to a very limited–and only more or less empirical–prediction of potential for further development in an intervention scenario. Biomarkers capable of categorizing training response would also be useful for medical purposes, especially with respect to the prediction of possible adverse developments in the cardiovascular and muscular systems. This study thus focuses on locating suitable sets of circulating miRNA biomarkers which would reflect the quality of the physiological process of physical exercise adaptation response. To achieve this, we investigated whether the change of selected extracellular miRNA levels is predictive of good or bad response to specific training programs: explosive strength (EXPL), hypertrophic strength training (HYP) and high-intensity interval training (HIIT), and whether it correlates with anthropometry and fitness parameters in a group of 30 subjects.

## Materials and methods

### Study participants

The study group included 30 young male athletes (mean age: 22.5 ± 4.06 years) recruited at the Faculty of Sports Studies of Masaryk University. All participants enrolled in this study were unrelated healthy Czechs of white European origin. Written informed consent was obtained and archived prior to participation in the training program and biological sample collection. The study was approved by the Ethics Committee of the Faculty of Sport Studies of Masaryk University (Brno, Czech Republic). Study participants were randomly assigned to three intervention sub-cohorts: explosive strength training (EXPL), hypertrophic strength training (HYP) and high-intensity interval training (HIIT) and subject to an 8-week-long exercise program. At specific time points during the training program (week 0, week 5 and week 8), blood plasma samples were collected, and anthropometric and physical activity parameters were measured.

At the beginning of the study, participants underwent a series of pre-tests and subsequently followed one of the intervention scenarios. After the completion of the intervention period, the same set of tests was performed. Pre-tests and post-tests were performed after more than 48 hours of non-intensive activity. Between two strength tests, i.e. vertical jump and isokinetic knee extensor test, the participants rested for three to five hours. Before the pre-tests, the participants attended two induction sessions and an initial diagnosis for exercise intensity determination. Based on these pre-analyses, all participants were found to be capable of performing the defined exercise without any limitations. The pre-test and post-test measurements included the testing of body composition, vertical jump and isokinetic and isometric knee extensor strength.

Anthropometric characteristics evaluated in this study were measured in light indoor clothes and without shoes using InBody720 (BIOSPACE, Korea), height was measured using a calibrated stadiometer. Immediately after body composition measurement subjects underwent a test of leg explosive strength by countermovement jump (CMJ). CMJ was measured as jump height using Pro2 (Myotest SA, Sion, Switzerland). Each strength test was performed as three subsequent CMJs with a 120 s recovery interval between individual jumps using the best result as a final outcome. Leg strength was analyzed using an isokinetic dynamometer Humac Norm CSMI (Stoughton MA, USA) with specific focus on the bilateral testing of knee extensors. Testing was performed using concentric mode with a 90° knee motion range and angular velocities of 60°/s and 300°/s. Six sub-maximal attempts with gradual strength increasing expression intended to muscle warm-up were performed prior to the actual test. Five maximal attempts were performed after a 30 s rest interval. Participants remained at rest for 180 s between various angular velocity measurements. Subsequently, isometric testing focusing on identifying the strength of knee extensors at a 90° angle was performed, i.e. participants performed one sub-maximal warm-up attempt followed by five maximal attempts with contraction duration of 5 s with 5 s rest intervals between individual measurements.

After 8 weeks of exercise, pre- and post-intervention characteristics were compared and participants were further separated into responders (RES) and non-responders (NRES). RES and NRES were individuals whose progress in selected characteristics was higher or lower than 5%, respectively. RES/NRES status was established for the following characteristics: 300°/s right leg extension (t300), 60°/s right leg extension (t60), vertical jump, right leg isometric extension (IE) and body fat percentage (BFP). All evaluated parameters are listed in **Table 1**.

**Table 1:** Participant characteristics during individual interventions at weeks 0, 5 and 8.

				intervention HIIT				intervention–explosive strength				Intervention–hypertrophic strength			
				time point (week)			intergroup	time point (week)			intergroup	time point (week)			intergroup
				0	5	8	comparison	0	5	8	comparison	0	5	8	comparison
Parameter		Unit	n	10	10	10	p-value*	10	10	10	p-value*	10	10	10	p-value*
Weight		kg		80.6 ± 7.8	81.3 ± 7.3	80.6 ± 7.1	0.441	74.7 ± 3.1	75.7 ± 3.2	75.8 ± 3.2	0.050	73.7 ± 8.8	74.6 ± 8.4	74.9 ± 8.5	0.004
Total body skeletal muscle mass (SMM)		kg		40.8 ± 3.2	41.2 ± 3.1	40.7 ± 2.8	0.273	38.3 ± 2.3	39.4 ± 2.2	39.4 ± 2.2	0.011	38.7 ± 4.9	39.4 ± 5.1	39.0 ± 5.1	0.140
Percentage of total body skeletal muscle mass (pSMM)		kg		50.8 ± 3.3	50.9 ± 3.7	50.7 ± 3.0	0.838	51.3 ± 2.3	52.1 ± 2.3	52.0 ± 2.1	0.080	52.5 ± 2.0	52.7 ± 2.4	52.0 ± 2.2	0.062
Right leg skeletal muscle mass (RLSMM)		kg		11.6 ± 0.9	11.6 ± 1.0	11.4 ± 1.0	0.395	10.4 ± 1.0	10.5 ± 0.9	10.5 ± 1.1	0.758	10.6 ± 1.5	10.7 ± 1.5	10.7 ± 1.5	0.805
Total body fat mass (FM)		kg		9.5 ± 5.5	9.5 ± 5.6	9.6 ± 4.9	0.931	7.8 ± 2.9	7.2 ± 2.9	7.3 ± 2.7	0.347	6.0 ± 2.6	6.0 ± 3.0	6.9 ± 2.9	0.033
Percentage of total body fat mass (pFM)		%		11.4 ± 5.7	11.4 ± 6.0	11.6 ± 5.2	0.841	10.4 ± 3.7	9.4 ± 3.8	9.7 ± 3.6	0.273	8.2 ± 3.2	8.2 ± 3.9	9.3 ± 3.6	0.044
300° right leg extension (t300)		N.m		119.8 ± 21.4	117.0 ± 19.5	120.4 ± 16.9	0.738	104.6 ± 9.5	107.7 ± 13.6	110.7 ± 12.4	0.020	107.2 ± 18.2	113.7 ± 18.1	117.1 ± 21.6	0.070
60° right leg extension (t60)		N.m		216.7 ± 40.1	219.7 ± 41.0	218.8 ± 34.6	0.820	206.5 ± 23.1	206.6 ± 24.2	209.9 ± 31.4	0.784	206.3 ± 55.9	209.4 ± 33.1	221.5 ± 38.6	0.236
Right leg isometric extension (IE)		N.m		248.9 ± 50.3	258.9 ± 63.4	269.5 ± 56.6	0.043	250.4 ± 36.8	257.2 ± 38.1	261.7 ± 33.7	0.502	269.7 ± 61.5	271.1 ± 50.0	308.0 ± 85.8	0.027
Vertical jump		cm		36.1 ± 6.5	38.5 ± 6.4	37.6 ± 6.2	0.014	35.9 ± 3.2	37.0 ± 4.1	37.4 ± 3.0	0.333	39.6 ± 3.2	39.9 ± 4.7	41.0 ± 4.5	0.242
miR-21		40—Ct values		7.3 ± 3.1	9.6 ± 1.8	7.5 ± 1.6	0.097	9.0 ± 2.9	8.1 ± 1.3	7.9 ± 1.1	0.623	6.5 ± 4.0	9.6 ± 2.4	7.8 ± 0.9	0.115
miR-222		40—Ct values		10.0 ± 0.7	10.1 ± 1.0	9.1 ± 1.2	0.143	10.4 ± 0.8	9.2 ± 1.0	9.2 ± 0.9	0.010	10.0 ± 1.1	10.6 ± 1.3	9.0 ± 1.1	0.018
miR-16		40—Ct values		17.9 ± 0.8	17.2 ± 2.4	15.0 ± 1.7	0.061	18.3 ± 1.1	15.5 ± 2.1	14.5 ± 0.7	0.001	17.8 ± 1.1	17.9 ± 2.3	14.6 ± 1.1	0.008
miR-93		40—Ct values		11.6 ± 1.8	12.0 ± 2.3	9.6 ± 2.0	0.027	11.2 ± 1.0	10.5 ± 2.1	9.7 ± 1.2	0.119	10.3 ± 1.1	12.8 ± 2.7	9.3 ± 1.4	0.004

*Presented p-value represents results from Friedman ANOVA or RMANOVA for each type of intervention at three selected time points

### Training interventions

Interventions were performed three times a week for a total of 8 weeks with a 48 h interval between individual training units. The actual intervention was performed after 5 minutes of warm-up at low intensity followed by 5 minutes of standard dynamic stretching between 8 to 10 AM.

The EXPL scenario included four sets of barbell jump squats with eight reps in each set. The knee bend was performed to 90% in the knee joint, with exercise intensity set to 90% of maximal power (Pmax). Rest intervals between sets were 3 minutes each. The HYP scenario consisted of barbell back squats and leg presses. Both exercises were performed in the full range of motion in three sets, i.e. total of 6 sets in one training unit, with 60 s rest intervals between the sets and individual exercises. Exercise intensity was set to 75%. The HIIT scenario encompassed the total of 30 running segments lasting 15 s each with 15 s rest intervals. The rest was passive, i.e. standing. Load intensity was set as running speed at 90% of maximum heart rate.

### miRNA analysis

#### Plasma RNA isolation

Blood samples were processed for plasma within one hour after collection by centrifugation at 2,000x g at 4°C for 20 min. Plasma was stored at -70°C, median storage time from collection to endpoint analysis was 12 months. Total RNA enriched for miRNAs was isolated from 200 μl of blood plasma samples using the miRNeasy Serum/Plasma Kit (Qiagen, Hilden, Germany) according to the manufacturer’s altered instructions– 1.25 μl of MS2 RNA (0.8 μg/μl) was added to QIAzol (Qiagen, Hilden, Germany). Isolated RNA purity and concentration were assessed by measuring its optical density (A260/A280 > 2.0; A260/A230 > 1.8) using the NanoDrop 1000 Spectrophotometer (Thermo Fisher Scientific, MA, USA). Isolated RNA samples were either stored at -70°C or further processed.

#### Plasma microRNA expression quantification by qRT-PCR

In the present study, we measured four circulating miRNA (c-miRNA) levels whose selection was based on their previous association with physical activity, i.e. miR-16-5p, miR-21-5p, miR-93-5p, and miR-222-3p [13,14]. Individual miRNA levels were determined by qRT-PCR consisting of two consequent steps. First, we performed reverse transcription according to the manufacturer’s protocol using the TaqMan® MicroRNA Reverse Transcription Kit (#4366596, Thermo Fisher Scientific, MA, USA) and miRNA-specific primers (#4427975, Thermo Fisher Scientific, MA, USA) and T100™ Thermal Cycler (Bio-Rad, CA, USA). After cDNA synthesis, we performed quantitative PCR according to the manufacturer’s protocol using TaqMan® Universal Master Mix II, no UNG (#4440040, Thermo Fisher Scientific, MA, USA), TaqMan® microRNA Assays (hsa-miR-16-000391, hsa-miR-21-000397, hsa-miR-93-5p-001090 and hsa-miR-222-002276, Thermo Fisher Scientific, MA, USA) and 7500 Real-Time PCR System (Thermo Fisher Scientific, MA, USA). miRNA levels were determined in duplicate and average threshold cycle (Ct) and standard deviation (SD) values were calculated. Final concentrations were expressed as the 40-Ct values as used previously [15].

#### Validated microRNA targets

Validated target genes of miRNAs which were differently expressed between responders and non-responders were analyzed using the online bioinformatic tool miRTarBase (miRTarBase.mbc.nctu.edu.tw/), release 6.0 [16]. Selected miRNA targets investigated in this study are listed in **Table 2**.

**Table 2:** Selected target mRNAs of candidate miRNAs.

miRNA	targeted gene function	target genes
miR-16	proliferation, differentiation, cell cycle regulation	*AKT3; AURKB; BRCA1; CCND1; CCND3; CCNE1; CCNT2; CDK6; CDS2; CHEK1; FGFR1; HDGF; IGF1R; KDR; MTOR; PDCD4; PIM1; PRDM4; PTGS2; PURA; RAF1; RICTOR; RPS6KB1; TP53; WEE1; YAP1*
	apoptosis	*BCL2; BIRC5; IGF1R; KDR; PIM1; RAF1; TP53*
	tissue remodeling, angiogenesis	*RECK; VEGFA; VEGFR1; VEGFR2*
	inflammation	*PTGS2*
	hematopoiesis	*MYB*
miR-21	proliferation, differentiation, cell cycle regulation	*BTG2; CDC25A; CDK2AP1; DUSP10; E2F1; E2F2; EGFR; IL1B; JMY; MEF2C; MYC; PDCD4; PPARA; PTEN; PTX3; RASA1; SASH1; SP1; STAT3; TGFBR2; TP53BP2; YOD1*
	apoptosis	*APAF1; BCL2; DAXX; FASLG; IGF1R; IL1B; MYC; SASH1; STAT3; TNFAIP3; TNFRSF10B; TP53BP2*
	tissue remodeling, angiogenesis	*HIF1A; MMP2; MMP9; PLAT; RECK; TIMP3; VEGFA*
	inflammation	*CCL20; CCR1; HPGD; IL1B; PPARA; PTX3*
	muscle contraction	*TPM1*
	antioxidant	*SOD3*
	glucose transport	*MAP2K3*
miR-93	proliferation, differentiation, cell cycle regulation	*CDKN1A; E2F1; EREG; MAPK9; MXD1; MYC; PTEN; PURA; SASH1; TGFB1; TGFBR2; WNT2B*
	apoptosis	*ATG16L1; HIF1A; MXD1; MYC; SASH1*
	tissue remodeling, angiogenesis	*HIF1A; VEGFA*
	inflammation	*IL8; MSK2*
	mitochondrial function	*TP53INP1*
	glucose transport	*SLC2A4; TRIP10*
miR-222	proliferation, differentiation, cell cycle regulation	*CDKN1B; CDKN1C; CERS2; CORO1A; DIRAS3; ETS1; FOS; HIPK1; HIPK2; KIT; PPP2R2A; PTEN; TP53; TP53BP2*
	apoptosis	*BBC3; CORO1A; ETS1; FOS; FOXO3; STAT5A; TNFSF10; TP53; TP53BP2*
	tissue remodeling, angiogenesis	*ETS1; KIT; MMP1; RECK; TIMP3*
	mitochondrial function	*SOD2*
	heart contraction	*GJA1*
	RNA interference	*DICER1*

### Statistical analysis

Statistical analysis was performed using the R program, v. 3.1.2. The normality of data distribution was tested using the Shapiro–Wilk normality test. The non-parametric Friedman test was performed to compare miRNA, functional parameters, anthropometry and the response status of individuals. Differences in other variables were compared using repeated measures ANOVA. When appropriate (*p* value < 0.05), a Dunn multiple comparison (miRNAs) or a Bonferroni multiple comparison (other variables) *post hoc* test was used to compare groups at different time points. Each result labeled with *p* < 0.05 indicates *p* values that resulted from the *post hoc* test. Correlations of miRNA profiles between baseline and other time points (5w, 8w) were calculated using the Pearson correlation analysis and correlations of miRNAs and anthropometry as well as functional characteristics were performed using Spearman rank correlation analysis as appropriate for the data distribution.

Univariate logistic models were constructed for responder status for each type of intervention, using investigated miRNAs separately as independent variables. Consequently, multivariate logistic models were constructed using responder status in selected tests as dependent variable and age, total skeletal muscle mass (in kg), miRNAs and type of intervention as independent variables. These models did not include total body mass as independent variable, as there was almost 100% correlation of total body mass and skeletal muscle mass.

The results are expressed as mean values ± standard deviations unless stated otherwise. Values of p < 0.05 and 0.05 < p < 0.10 were considered statistically significant and borderline significant respectively.

## Results

### Participant characteristics

No differences in anthropometric parameters were observed between investigated groups of subjects at the beginning of the study. With respect to physical activity parameters, a significant difference in the vertical jump was observed between the groups at baseline (vertical jump: HIIT vs EXPL vs HYP, 36.1 ± 6.5 vs 35.9 ± 3.2 vs 39.6 ± 3.2, p = 0.032). Therefore, the vertical jump and all parameters derived from it (i.e. performance, force and acceleration) are not further discussed. Of the original cohort, a total of 30 participants successfully completed the exercise interventions (HIIT n = 10, EXPL n = 10 and HYP n = 10) and were therefore included in the final analysis. One participant was excluded due to missing data. The flowchart describing the evolution of the study is given as **S1 Fig**. The training progress of the participant during the study is shown in **Table 1**. Various degrees of change were observed in individual intervention groups. We observed (1) a significant increase in IE, vertical jump, performance and force, (2) a significant increase in weight, SMM, pFM and t300 and a borderline significant increase in pSMM and force, and (3) a significant increase in weight, FM and IE and a borderline significant increase in pFM, t300 and pSMM in the HIIT, EXPL and HYP intervention groups respectively.

### Plasma microRNA level dynamics during interventions

**Fig 1:**
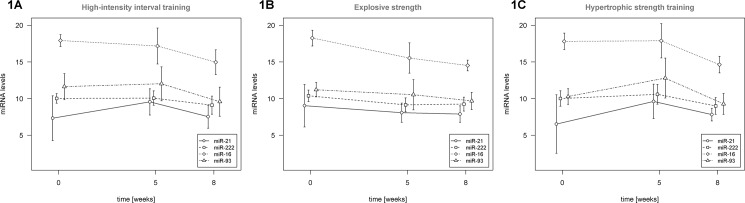
Dynamics of investigated miRNA levels throughout the study period–HIIT intervention, explosive and hypertrophic strength training.

In the HIIT intervention scenario, miR-21 (p = 0.097) and miR-93 (p = 0.027) levels increased after 5 weeks of exercise and then dropped to below the initial level after 8 weeks of exercise, while miR-16 (p = 0.061) decreased gradually (**Fig 1A**). In the EXPL intervention scenario, we observed a drop in miR-222 (p = 0.01) level after 5 weeks of exercise, which then remained stable until the end of the intervention program (**Fig 1B**). A gradual decrease was observed in the case of miR-16 (p = 0.001) level. During the third intervention type, i.e. HYP exercise, we observed an overall increase in miR-93 (p = 0.004), miR-16 (p = 0.008) and miR-222 (p = 0.018) after 5 weeks of intervention and a subsequent decrease to below the initial miRNA level after the completion of the exercise program (**Fig 1C).**

### Relationship between candidate miRNAs and different intervention types

No differences in miRNA levels were apparent between the intervention groups at baseline. No statistically significant prediction role was observed using crude univariate stepwise regression model analysis where RES/NRES status for t300, t60, IE, vertical jump and pFM was used as a dependent variable and miR-21, miR-222, miR-16 and miR-93 levels at baseline were used as independent variables. Likewise, no significant association was observed using multivariate stepwise regression model analysis adjusted for intervention type, age, SMM and miR-21, miR-222 and miR-16 levels at baseline with RES/NRES status for t300, t60, IE, vertical jump and pFM used as a dependent variable. However, the baseline levels of miR-93 expressed an independent prediction role for responder status based on isometric extension of the right leg (beta estimate 0.76, 95% CI: -0.01; 1.53, p = 0.052). The results of the multivariable modeling are given in **Table 3**.

**Table 3:** Results of the multivariable regression modeling of responder status for given miRNAs.

**miR16**					
Variable	beta estimate	95% CI for beta estimate	OR estimate	95% CI for OR estimate	p value
t300 R:N	0.21	(-0.63; 1.06)	1.24	(0.53; 2.87)	0.619
t60 R:N	0.12	(-0.74; 0.97)	1.12	(0.48; 2.63)	0.791
IPE R:N	0.40	(-0.44; 1.24)	1.49	(0.64; 3.45)	0.354
jump R:N	0.23	(-0.63; 1.08)	1.25	(0.54; 2.94)	0.603
% body fat R:N	0.63	(-0.43; 1.68)	1.87	(0.65; 5.38)	0.243
**miR21**					
Variable	beta estimate	95% CI for beta estimate	OR estimate	95% CI for OR estimate	p value
t300 R:N	-0.03	(-0.28; 0.21)	0.97	(0.76; 1.24)	0.802
t60 R:N	-0.16	(-0.42; 0.1)	0.85	(0.66; 1.10)	0.227
IPE R:N	0.09	(-0.15; 0.33)	1.09	(0.86; 1.39)	0.469
jump R:N	0.19	(-0.08; 0.45)	1.21	(0.93; 1.57)	0.165
% body fat R:N	0.27	(-0.09; 0.63)	1.31	(0.91; 1.87)	0.142
**miR222**					
Variable	beta estimate	95% CI for beta estimate	OR estimate	95% CI for OR estimate	p value
t300 R:N	0.18	(-0.79; 1.15)	1.20	(0.45; 3.16)	0.716
t60 R:N	-0.07	(-1.1; 0.95)	0.93	(0.33; 2.58)	0.886
IPE R:N	0.33	(-0.69; 1.34)	1.39	(0.50; 3.82)	0.529
jump R:N	0.33	(-0.7; 1.36)	1.39	(0.50; 3.88)	0.527
% body fat R:N	0.90	(-0.28; 2.08)	2.46	(0.76; 8.00)	0.134
**miR93**					
Variable	beta estimate	95% CI for beta estimate	OR estimate	95% CI for OR estimate	p value
t300 R:N	0.27	(-0.45; 0.98)	1.31	(0.64; 2.68)	0.464
t60 R:N	-0.19	(-0.86; 0.47)	0.82	(0.42; 1.60)	0.570
IPE R:N	0.76	(-0.01; 1.53)	2.14	(0.99; 4.60)	0.052
jump R:N	0.24	(-0.39; 0.87)	1.27	(0.68; 2.39)	0.453
% body fat R:N	0.23	(-0.55; 1.02)	1.26	(0.58; 2.76)	0.560

Several positive associations were observed when a linear mixed effect model with autoregressive covariant structure, which takes into account the interaction of intervention type, time and RES/NRES status for t300, t60, IE, vertical jump and pFM, was employed. In the case of RES/NRES for IE, we observed a significant association with miR-222 (p = 0.035) and miR-16 (p = 0.022) and a borderline significant association with miR-93 (p = 0.071). In the case of RES/NRES status for pFM, a significant association was observed with miR-16 (p = 0.008) and a borderline significant association with miR-222 (p = 0.081) and miR-93 (p = 0.093). Thus, using this model, we were able to distinguish individual interventions in the RES groups for IE and pFM parameters based on miRNA levels. For the IE parameter: (1) miR-16 levels were significantly different between responders to HIIT and EXPL (p = 0.002) and between HYP and EXPL (p = 0.005); (2) miR-222 levels were significantly different between responders to HIIT and EXPL (p = 0.088) and between HYP and EXPL (p = 0.004); and (3) miR-93 levels were significantly different between responders to HIIT and EXPL (p = 0.014) and between HYP and EXPL (p = 0.064). For the pFM parameter: (1) miR-16 levels were significantly different between responders to HIIT and HYP (p = 0.028); and (2) miR-222 levels were significantly different between responders to HIIT and HYP (p = 0.036).

### Verified targets of altered c-miRNAs

**Fig 2:**
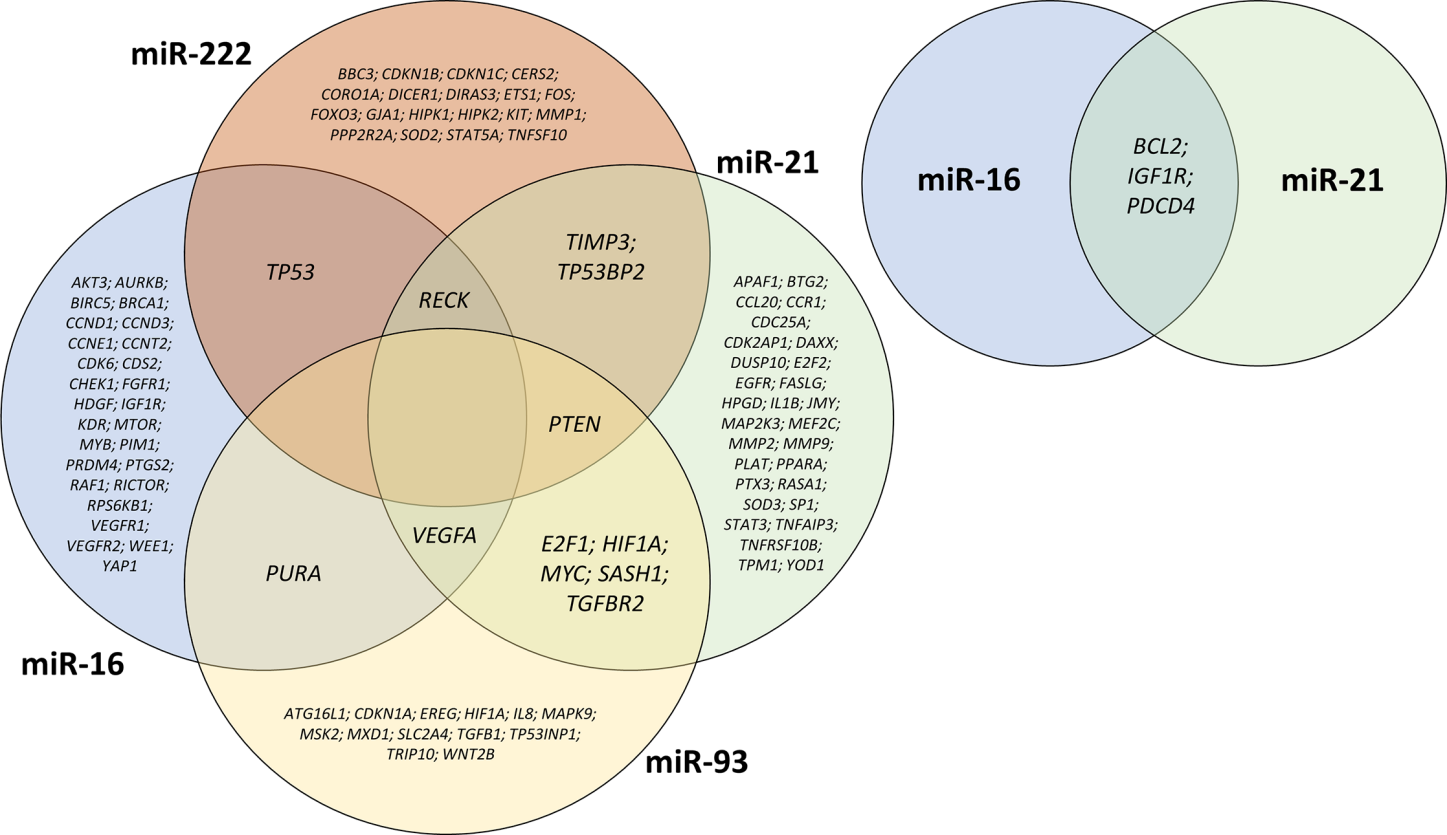
Venn diagram of common and different targets of investigated miRNAs.

Using miRTarBase, we identified 611 verified target genes for miR-16, 377 for miR-21, 1,527 for miR-93 and 1,190 for miR-222. These results were subsequently narrowed down to targets verified by strong evidence-based methods (i.e. reporter assay, Western blot and qPCR). Finally, we assessed 60 verified target genes for miR-16, 113 for miR-21, 35 for miR-93 and 41 for miR-222. Of these, we further selected genes linked to exercise-induced physiological adaptations (i.e. muscle growth, mitochondrial synthesis, angiogenesis, etc.), see **Table 3**, for the common and different targets see also **Fig 2**.

## Conclusions

The several reviews have recently summarized the role of circulating miRNAs in adaptation to physical activity [17–19] and all conclude that the specific c-miRNAs are altered in response to various protocols of exercise among highly-trained individual, typically healthy adults, as well as in response to specific diseases. However, very little information is available on the actual differences in miRNA expression between the exercise protocols of specific types of interventions. This study shows that miR-16, miR-21, miR-222 and miR-93 are down- or upregulated during explosive strength (EXPL), hypertrophic strength training (HYP) and high-intensity interval training (HIIT).

Traditionally, various hormones and anthropometric and physiological variables have been used to assess the response to a given type of exercise. However, such monitoring has its limitations–the hormonal response tends to be very time-limited and difficult to interpret, anthropometric variables tend to change over rather long intervals and physiological variables are rather crude and non-specific for a given type of exercise program. Thus, since traditional approaches do not consider the functional and subsequent structural remodeling of tissues in the context of extra- and intracellular signaling pathways, novel and more specific biomarkers of response to exercise are clearly needed. These biomarkers should be present in the bloodstream to facilitate measurement while also being relatively stable over time, which makes c-miRNAs very good candidates.

MiR-16 is an important player in the reduction of proliferation, migration and angiogenic behavior in endothelial cells (ECs) [20]. It has been recently reported that physical activity in spontaneous-hypertensive rats lead to a reduction of miR-16 and miR-21 levels and on the contrary, to elevated vascular endothelial growth factor and Bcl-2 levels [20]. MiR-16 regulates cell-autonomous angiogenic functions in ECs by targeting VEGFR2 and FGFR1 and its overexpression leads to reduced proliferation, migration and cord formation of ECs in vitro.[21] Wahl et al. suggest that an acute bout of exercise seems not to affect c-miR-16 in trained athletes, which supports the hypothesis that the upregulation of other c-miRNAs like c-miR-21 or c-miR-126 results in a switch of the ratio of these miRNAs, which may in turn be responsible for the observed effects [22]. In this study we observed a significant reduction of miR-16 levels in all three interventional arms, which is well in line with animal experiments conducted by Fernandes et al. [20]. Based on our results, all three exercise scenarios led to a reduction of miR-16, suggesting that the effect is rather non-specific.

Generally speaking, miR-21 is an elegant candidate for the assessment of response to physical exercise as it is indirectly involved in angiogenesis by inducing hypoxia inducible factor-1 (HIF-1α) and VEGF expression as well as regulating apoptosis, increasing endothelial nitric oxide synthase activity and mediating anti-inflammatory response in macrophages [23]. Wahl et al. hypothesize that especially during all-out exercise bouts, local short-term and transient hypoxia occurs, which could explain the increases of c-miR-21 after specific types of exercise [22]. In this study, we did not observe any sustained response of miR-21 to any type of intervention throughout the entire 8 weeks of the study in any exercise scenario. This corresponds to previous observations which indicate that this c-miRNA is responsive to acute exercise before but not after sustained training [24]. On the other hand, Nielsen et al. report the miR-21 circulating levels decrease after 12 weeks of chronic training but report no effect of 1 h of cycling at 65% Pmax on c-miR-21 in well-trained subjects. However, it is important to note that Nielsen et al. focused on endurance training which was not investigated in our study. In a recent study of three different exercise protocols, specifically muscular strength endurance (SE), muscular hypertrophy (MH) and maximum strength (MS), Cui et al. reported that miR-21 decreases immediately after exercise, followed by an increase during early recovery, which could be indicative of its important role in balancing pro-inflammatory and subsequent immunoregulatory anti-inflammatory processes [25]. A recent study by Wahl et al. [22] investigated the effect of different exercise protocols including HIIT on the miR-16, miR-21, and miR-126, HIT was associated with only small to moderate changes on c-miRs-21 (Cohen’s d = -0.28).

Evidence provided by animal models and in vitro studies suggest that miR-222 is indispensable for exercise-induced cardiac growth and proliferation in the adult mammalian heart and also plays a vital role in the protection of the heart against adverse remodeling following ischemic injury [26]. Potential miR-222 targets involved in the regulation of cardiac function include cell cycle inhibitor p27 (CDKN1B) and kinases HIPK1 and HIPK2 [26]. Moreover, additional cells such as vascular smooth muscle cells and ECs are also known contributors to circulating miR-222 levels. The regulative role of miR-222 is at least partially mediated by stem cell factor (SCF) receptor, c-Kit. A decline in c-Kit leads to impairment in endothelial cell migration, proliferation and angiogenesis [27]. In the case of miR-222, we observed a significant decrease in c-levels after 5 weeks of training protocol in the explosive strength group, but not in hypertrophic strength training and HIIT groups. This contradicts a study by Baggish et al. who report an increase, not a decrease, after chronic exercise [24]. Also, the baseline miR-222 levels cited by Baggish were significantly lower than in our study (2.40 ± 0.42-fold change vs. 10.0 ± 0.7 in our study), which could be attributed to slightly different subject characteristics at baseline (differences in age and overall fitness) as well as to different types of training modalities employed by each of the two studies. Similarly to the results reported by Baggish, levels of ci miR-222 also increased following cardiopulmonary exercise in a cohort of heart failure patients [26]. Interestingly, in a study by Bye et al., miR-222 and miR-21 levels were not correlated with any exercise habits in terms of frequency, intensity or duration of self-reported regular exercise training. Moreover, a strong correlation of miR-222 and miR-21 was found for aerobic fitness measured as maximal oxygen uptake. Subjects with low aerobic fitness were found to have higher circulating levels of both miR-222 and miR-21 [28]. In a study by Wardle et al. miR-222 was present at different levels in three participant groups (p = 0.028) with the highest levels being observed in the endurance scenario and the lowest in the hypertrophic strength training scenario (miR-222 being 1.94 times higher in endurance exercise subjects (95% CI, 1.73–2.18) [14]. These differences were not observed in this study, possibly due to the better overall fitness of subjects employed by Wardle et al.

MiR-93 was previously characterized as a signature miRNA in diabetes and a critical regulator of VEGF-A expression [29]. MiR-93 is capable of inducing changes in chromatin structure via H3S10P through mitogen- and stress-induced kinase 2 (Msk2), which serves as a kinase to phosphorylate H3 at Ser10, among other targets [30]. The upregulation of Msk2 in exercising muscle has been known for a longer time; however, the exact relationship between Msk2 upregulation in skeletal muscle and the type of exercise scenario is currently unknown. It may be suggested that miR-93 is a key player in the regulation of Msk2 expression. In the case of miR-93, we also observed a significant tendency towards the reduction of its levels in connection with the ongoing exercise scenario in all three interventional groups.

As already mentioned, we investigated in this study whether the selected set of miRNAs is associated with response to explosive strength, hypertrophic strength training and high-intensity interval training. We observed reduction of miR-16 and miR-93 in all study arms, decrease of miR-222 in the explosive strength group and only insignificant differences in miR-21 levels between the study arms. The very low response of c-miR-21 levels is not surprising as the study arms were based mainly on resistance training, and higher response of c-miR-21 would be expected rather in endurance training. However, it is very difficult to find the biologically plausible explanation for the reduction of miR-16 levels in all three study arms on one hand and simultaneously no differences in miR-21 levels as both of these miRNAs target the VEGF/VEGFR pathway that is involved in adaptation to different types of physical activity. The same applies to miR-93, an important regulator of VEGF-A that was consistently reduced in all three study arms. Considering the functional pathway of miR-93 working via the Msk2 that is activated by muscle contraction, it seems that the signaling cascades within the working muscle are activated rather locally than systemically. However, more research into the exact mechanisms of functioning of VEGF pathway, Msk2 and associated miRNAs in working muscle is necessary before any robust conclusions can be derived on their mechanistic link.

To conclude, we decided for the candidate approach in this study instead of the whole-genome high-throughput approach. We selected four candidate microRNAs based on the literature search and their potential functional link with biological processes related to different training scenarios and identified multiple associations. We believe that our exploratory study provides rationale for the whole-genome high-throughput approach based on RNAseq to obtain more comprehensive picture on circulating microRNAs related to different training programs in the future.

### Limitations

The presented study has specific limitations. First, the participants in the present study had to maintain a regular exercise regimen for 8 weeks. While compliance with the study protocol was empirically tested several times during the study period, the same compliance of the study subjects with the tested protocols cannot be taken for granted. As the study subjects were active athletes, they entered the study in a pre-trained state with significant interindividual variability in terms of their physical fitness. Even though the study subjects were randomly assigned to the intervention sub-cohorts of the study, it may be possible for the baseline characteristics of study subjects to differ between them. Furthermore, the 8-week-long period may be too short to induce measurable differences between groups. The number of participants in the specific sub-cohorts of this intervention study was low and larger cohorts are clearly needed to confirm our observations.

### Strengths

This study constitutes the first interventional study focused on a comparison of response to 8-week-long explosive strength training (EXPL), hypertrophic strength training (HYP) and high-intensity interval training (HIIT) with selected extracellular miRNAs.

### Conclusion

In summary, our data demonstrate that each investigated exercise scenario was associated with distinct functional and structural features of the subjects and by sustained trends in investigated c-miRNA levels. In accordance with previous reports, our study indicates that circulating miRNAs can be significantly altered by physical activity. More specifically, the findings of the present study indicate that 8-week-long explosive strength (EXPL) training, hypertrophic strength training (HYP) and high-intensity interval training (HIIT) regimens are associated with significant changes in miR-16, mir-21, miR-222 and miR-93 levels compared to a baseline in athletic young men. The relatively large statistical effects on relatively small cohorts of subjects might be indicative of miRNAs being actively involved in exercise-induced anthropometric as well as fitness phenotype changes.

## Supporting information

S1 Fig A flowchart describing the evolution of the study. (DOCX)

S1 Table The raw data used for analyses. (XLSX)

## Abbreviations

miRNA: microRNA
RES: responders
NRES: non-responders
SMM: skeletal muscle mass
pSMM: percentage of total body skeletal muscle mass
RLSMM: right leg skeletal muscle mass
FM: total body fat mass
pFM: percentage of total body fat mass
t300: 300°/s right leg extension
t60: 60°/s right leg extension
IE: sometric extension
CMJ: countermovement jump
EXPL: explosive strength training scenario
HYP: hypertrophic strength training scenario
HIIT: high-intensity interval training
