# Supplementary material for: Exercise-induced circulating microRNA changes in athletes in various training scenarios
Source: PLoS One. 2018 Jan 16;13(1):e0191060. doi: 10.1371/journal.pone.0191060 (PMC5770042; doi:10.1371/journal.pone.0191060)
Supplement: S1 Fig — (DOCX) [file pone.0191060.s001.docx]

S1 Fig: **A flowchart describing study evolution.** 300°/s right leg extension (t300), 60°/s right leg extension (t60), isometric extension (IE), vertical jump, isometric extension of the right leg and body fat percentage (BFP).

- Selection of candidate miRNAs
- Training intervention programs design

Study results

- MicroRNA analysis (n = 30)
- Statistical analysis

Study completion

Completed: n = 10

Completed: n = 10

Completed: n = 10

Separation into responders (RES) and non-responders (NRES) according to following parameters: t300; t60; IE; vertical jump; right leg extension; BFP.

(NRES ≤ 5 % ˂ RES)

Final sample collection and testing (week = 8)

Injury (n = 1)

Second sample collection and progress testing (week = 5)

Hight-intensity interval training – HIIT (n = 11)

Hypertrophic strength training – HYP (n = 10)

Explosive strength training – EXPL (N = 10)

Random distribution into intervention groups

Training intervention start (week = 0)

PRE-TESTING:

- Body composition measurement
- Strength and dynamometric tests
- Blood sample collection

Did not attend initial diagnosis: n = 2

- Initial diagnosis
- Exercise intensity determination

Recruitment of young athletes at the university (n = 33)
